# Effect of CO_2_ Concentration on Uptake and Assimilation of Inorganic Carbon in the Extreme Acidophile *Acidithiobacillus ferrooxidans*

**DOI:** 10.3389/fmicb.2019.00603

**Published:** 2019-04-04

**Authors:** Mario Esparza, Eugenia Jedlicki, Carolina González, Mark Dopson, David S. Holmes

**Affiliations:** ^1^Laboratorio de Biominería, Departamento de Biotecnología, Facultad de Ciencias del Mar y Recursos Biológicos, Universidad de Antofagasta, Antofagasta, Chile; ^2^Center for Bioinformatics and Genome Biology, Fundación Ciencia & Vida, Santiago, Chile; ^3^Centre for Ecology and Evolution in Microbial Model Systems (EEMiS), Linnaeus University, Kalmar, Sweden; ^4^Centro de Genómica y Bioinformática, Facultad de Ciencias, Universidad Mayor, Santiago, Chile

**Keywords:** CO_2_ fixation, CCM, carbon concentration mechanism, *Acidithiobacillus ferrooxidans*, acidic environment, low pH environment, bicarbonate uptake, RubisCO

## Abstract

This study was motivated by surprising gaps in the current knowledge of microbial inorganic carbon (Ci) uptake and assimilation at acidic pH values (pH < 3). Particularly striking is the limited understanding of the differences between Ci uptake mechanisms in acidic versus circumneutral environments where the Ci predominantly occurs either as a dissolved gas (CO_2_) or as bicarbonate (HCO_3_^-^), respectively. In order to gain initial traction on the problem, the relative abundance of transcripts encoding proteins involved in Ci uptake and assimilation was studied in the autotrophic, polyextreme acidophile *Acidithiobacillus ferrooxidans* whose optimum pH for growth is 2.5 using ferrous iron as an energy source, although they are able to grow at pH 5 when using sulfur as an energy source. The relative abundance of transcripts of five operons (cbb1-5) and one gene cluster (*can*-*sulP*) was monitored by RT-qPCR and, in selected cases, at the protein level by Western blotting, when cells were grown under different regimens of CO_2_ concentration in elemental sulfur. Of particular note was the absence of a classical bicarbonate uptake system in *A. ferrooxidans*. However, bioinformatic approaches predict that *sulP*, previously annotated as a sulfate transporter, is a novel type of bicarbonate transporter. A conceptual model of CO_2_ fixation was constructed from combined bioinformatic and experimental approaches that suggests strategies for providing ecological flexibility under changing concentrations of CO_2_ and provides a portal to elucidating Ci uptake and regulation in acidic conditions. The results could advance the understanding of industrial bioleaching processes to recover metals such as copper at acidic pH. In addition, they may also shed light on how chemolithoautotrophic acidophiles influence the nutrient and energy balance in naturally occurring low pH environments.

## Introduction

*Acidithiobacillus ferrooxidans* is a polyextremophile inhabiting very acidic (pH < 3) and often metal laden environments that belongs to the *Acidithiobacillia* class within the *Proteobacteria* (Williams and Kelly, 2013). It is an obligate chemolithoautotrophic, mesophilic microorganism that gains energy and reducing power by the aerobic oxidation of hydrogen, inorganic sulfur compounds, and ferrous iron (Bonnefoy and Holmes, 2012; Dopson and Johnson, 2012) and anaerobically via sulfur or formate oxidation coupled to reduction of ferric iron (Pronk et al., 1991; Hedrich and Johnson, 2013; Osorio et al., 2013).

*A. ferrooxidans* is one of the most abundant microorganisms found at ambient temperatures in industrial bioleaching heaps used for the recovery of, e.g., copper (Soto et al., 2013; Vera et al., 2013; Zhang et al., 2016). It also forms an integral part of natural occurring acidic ecosystems such as the Rio Tinto and deep subsurface in the Iberian pyrite belt (Amils et al., 2014), acidic springs, cave systems plus volcanic soils (reviewed in Johnson, 2012; Hedrich and Schippers, 2016), and acid mine drainage (AMD) (Chen et al., 2015; Teng et al., 2017). *A. ferrooxidans* is considered a model species for understanding genetic and metabolic functions reviewed in Cardenas et al., 2016) and survival mechanisms at extremely low pH (Chao et al., 2008) and reviewed in Slonczewski et al. (2009). It has also provided useful information for understanding how microorganisms can contribute to the nutrient and energy balance in bioleaching heaps (Valdes et al., 2008; Valdés et al., 2010).

The dominant source of available inorganic carbon (Ci) in circumneutral and slightly alkaline environments such as terrestrial fresh water and oceans is bicarbonate (HCO^-^_3_) with lower concentrations of dissolved CO_2_ (Mangan et al., 2016). The majority of models for prokaryotic Ci uptake and assimilation have been elucidated for organisms, such as cyanobacteria, that inhabit these environments (Burnap et al., 2015; Klanchui et al., 2017). Cyanobacteria fix carbon via the Calvin-Benson-Bassham (CBB) cycle and use a variety of carbon concentration mechanisms (CCMs) to take up CO_2_ or bicarbonate and provide CO_2_ to the carbon fixation enzyme, ribulose bisphosphate carboxylase-oxygenase (RubisCO). Five C_i_ uptake systems have been reported including three bicarbonate transporters: BCT1, SbtA, and BicA that vary in affinity and flux for bicarbonate and two intracellular CO_2_ “uptake” systems, that convert CO_2_, passively diffusing into the cell, into bicarbonate (Burnap et al., 2015; Klanchui et al., 2017). The transporters vary in affinity and flux for bicarbonate providing a selective advantage to organisms in environments with a wide dynamic range of HCO_3_^-^ availability. For example, freshwater β-cyanobacteria that live at about pH 7 not only use the high affinity SbtA transporter and the low affinity, high flux BicA transporter but also the medium affinity BCT1, an inducible bicarbonate transporter under limited Ci conditions (Sandrini et al., 2014, 2015; Klanchui et al., 2017). Alkaline lake β-cyanobacteria tend to have just BicA and it is hypothesized that the high affinity SbtA is not necessary in environments rich in HCO_3_^-^ (Klanchui et al., 2017).

In contrast, less is known about Ci uptake and assimilation in extremely acidic environments where the dominant source of Ci is the dissolved gas CO_2_ (Carroll and Mather, 1992; Cardenas et al., 2010; Valdés et al., 2010; Mangan et al., 2016; WikiVividly, 2018). *A. ferrooxidans* fixes carbon by the CBB cycle (Esparza et al., 2010). Bioinformatic analyses, EMSA assays, and complementation of mutants in the surrogate host *Cupriavidus necator* (formerly *Ralstonia eutropha*) have demonstrated the presence of four operons (cbb1-4) of CBB cycle genes in *A. ferrooxidans* that are involved in Ci uptake and assimilation. Operons cbb1-3 were shown experimentally to be regulated by CbbR, a LysR-family transcription regulator (Esparza et al., 2009, 2010, 2015). In the present study, RNA transcript and protein abundance profiles were determined for genes present in *A. ferrooxidans* operons cbb1-4 under different CO_2_ concentrations. In addition, a fifth cbb operon (cbb5) and a gene cluster predicted to encode a bicarbonate uptake transporter and a carbonic anhydrase were detected and were also evaluated for expression under different CO_2_ concentration regimes. Acquiring this knowledge is important considering the central roles that the CCM and CBB cycle genes play in the determination of CO_2_ fixation and biomass formation in extremely acidic environments.

## Materials and Methods

### Bacterial Strains and Culture Conditions

*A. ferrooxidans* ATCC 23270 was cultured in 9K medium (Quatrini et al., 2007) adjusted to pH 3.5 with H_2_SO_4_ and containing 5 g/L elemental sulfur at 30°C under aerobic conditions (0.036% CO_2_). Increased concentrations of CO_2_ were obtained by sparging with a mixture of CO_2_ and air by changing the ratio of CO_2_ in the gas mixture. *A. ferrooxidans* cultures were grown to mid-log phase (Guacucano et al., 2000) as measured by cell counts using a Neubauer chamber. Cells were rapidly cooled on ice and then centrifuged at 800 × *g* for 5 min at 4°C to remove solid sulfur particles followed by cell capture by centrifugation at 8,000 × *g* for 10 min at 4°C. The cell pellet was re-suspended in ice-cold 9K salt solution for further washing. Total RNA was prepared immediately after cell harvesting.

### Isolation of RNA and Real-Time Quantitative PCR (RT-qPCR) Assays

Total RNA was isolated from *A. ferrooxidans* cells as described previously (Guacucano et al., 2000). The RNA preparations were treated with DNase I (Fermentas) before proceeding with the cDNA synthesis step. One microgram of total cellular RNA was used for each reaction. Real-time quantitative RT-PCR (RT-qPCR) was performed using RevertAid H Minus Reverse Transcriptase (Fermentas). The sequences of the qPCR primers for genes involved in CO_2_ assimilation are provided in Table 1. Control reactions performed using RNA but lacking reverse transcriptase to assess genomic DNA contamination did not produce any bands after gel electrophoresis (data not shown). RT-qPCR assays were carried out in a 25 μL PCR mixture consisting of 12.5 μl 2 × SYBR Green Supermix (Bio-Rad). The RT- qPCR was performed on iCycler iQ Real-time PCR detection system (Bio-Rad Laboratories, United States) with IQ SYBR green supermix (Bio-Rad) as described previously (Liu et al., 2011). Quantification of the target gene expression was performed using iCycler iQ5^TM^ software using a normalized expression analysis method as described by the manufacturer. Relative quantifications were performed from duplicate biological replicates using expression of *rec*A as a control as described previously (Esparza et al., 2015). PCR primers were designed as described (Thornton and Basu, 2011) and the results were analyzed using IQ Bio-Rad equipment software RT-qPCR and excel software. Statistical variance was analyzed using the Tukey test (Tukey, 1949) and ANOVA (Kotz et al., 2014). The *P*-value was 0.05.

**Table 1:** PCR primers used in the study.

Gene	Forward primer 5′–3′	Reverse primer 5′–3′
*cbbR1*	TCAGCCGCCGGAACACATA	CAACGCCGTGTTGCTCGAA
*cbbM*	ATGACGAAATCCTCCCGGACC	CACGTTCAGGAGCAGCGCAT
*cbbS1*	GCATCGAGCATGTGGAGCCT	GCGGAACACCACAAAAGCG
*cbbS2*	TAGAACATACCGAACCGGAAAACG	GCCCCGATAGACTACCAGGGAAG
*can2*	CAATATCGCCAACCTCGTGCC	CGTCTTTGGCAATGTCCACCC
*rsmE*	ATCAGGCCCTCATTCTGCAGC	GATCCATCTGGCAGGTCACACC
*cbbG*	AGCACCACATCGTCTCCAACG	GCTGGTGGGGATCATGCTCAT
*cbbP*	AGACACCATCCTGCGCCGTAT	GCAGGAGGGTGGGGAAATTCT
*recA*	CACCGGTGGTAATGCCCTTAAAT	ACACCGAGGTCCACCAGTTCG

### Production of CbbR Antisera and Western Blotting

Antibodies against CbbR were obtained as previously described (Esparza et al., 2010). Antibodies against phosphoribulokinase (CbbP) and RubisCO small sub-unit (CbbS) were provided by Dr. Botho Bowien (Institut für Mikrobiologie und Genetik, Georg-August-Universität Göttingen, Germany). The Western Blotting was performed as previously described (Quatrini et al., 2005) using the Supersignal West Pico chemiluminescent substrate (Pierce).

### Bioinformatics Methods

Experimentally validated and predicted SulP protein sequences were obtained from multiple Bacteria including cyanobacteria (Price et al., 2004), sulfate-reducing microorganisms (Marietou et al., 2018), and other microorganisms (Moraes and Reithmeier, 2012). These included the experimentally validated sulfate transporter Rv1739c from *Mycobacterium tuberculosis* H37Rv (Marietou et al., 2018) and experimentally validated bicarbonate transporters YchM from *Escherichia coli* APEC O1 and *E. coli O157:H7* str. Sakai (Moraes and Reithmeier, 2012), Rv3273 from *M. tuberculosis* H37Rv (Felce and Saier, 2004), and BicA from *Synechococcus* sp. PCC 7002 (Price et al., 2004). Additional SulP protein sequences were added to the analysis (Ward et al., 2004; Boden et al., 2011; Huntley et al., 2011; Svenning et al., 2011; Scott et al., 2019). A multiple sequence alignment was constructed using MAFFT with accurate option L-INS-i as alignment tools (Katoh and Standley, 2013; Nakamura et al., 2018) and ClustalW alignment program (Larkin et al., 2007). The alignments were imported into GBLOCKS (Castresana, 2000) and phylogenetically uninformative or noisy, unreliable sections of the alignments were masked (Rajan, 2013). Resultant alignments were used to construct a maximum likelihood, unrooted, phylogenetic tree with IQtree (Nguyen et al., 2015) using 1000 replicates. Trees were inferred using the LG + I + G model with empirically determined amino acid frequencies according to the ProtTest (Darriba et al., 2011) tool. Trees were visualized and annotated in Figtree^1^.

Transmembrane regions in protein sequences were predicted with TMHMM (Sonnhammer et al., 1998; Krogh et al., 2001) and TMPRED (Hofmann, 1993). Subcellular localization for protein sequences was predicted using PSORTb (Yu et al., 2010). Gene functional associations were predicted using String (Szklarczyk et al., 2017). RNA Secondary structure was predicted using Mfold (Zuker, 2003).

A list of *A. ferrooxidans* genes used in this study, their predicted functions and GenBank locus tags is provided in Table 2. The table has been updated from Esparza et al. (2010).

**Table 2:** Genes, predicted functions, and GenBank locus tags for the *A. ferrooxidans* CBB cycle and CCM genes used in this study.

Gene	Predicted function	GenBank locus tag^a^
***cbb1* operon**		
*cbbR*	LysR family transcriptional regulatory protein	AFE_1692
*cbbL1*	Ribulose bisphosphate carboxylase large chain 1 [EC:4.1.1.39] RubisCO type I	AFE_1691
*cbbS1*	Ribulose bisphosphate carboxylase small chain 1 [EC:4.1.1. 39] RubisCO type IAc	AFE_1690
*csoS2*	Carboxysome structural peptide CsoS2	AFE_1689
*csoS3*	Can1, carbonic anhydrase, 𝜀-type	AFE_1688
*csoPA*	Carboxysome peptide A	AFE_1687
*csoPB*	Carboxysome peptide B	AFE_1686
*cso*S1B	Microcompartments protein	AFE_1685
*cso*S1B	Microcompartments protein	AFE_1683
*cso*S1B	Microcompartments protein	AFE_1684
*bfr*A	Bacterioferritin	AFE_1682
*hyp*1	Conserved hypothetical protein	AFE_1679
*par*A	Partition protein A	AFE_1675
*hyp*2	Conserved hypothetical protein, Pterin-4a-carbinolamine Dehydratase/Dimerization Cofactor family	AFE_1681
*cbb*Q1	RubisCO activation protein CbbQ1	AFE_1678
*cbb*O1	RubisCO activation protein CbbO1	AFE_1677
*cbb*A	Fructose-bisphosphate aldolase [EC:4.1.2.13]	AFE_1676
***cbb2* operon**		
*cbbL2*	Ribulose bisphosphate carboxylase large chain 1 [EC:4.1.1.39]	AFE_3051
*cbbS2*	Ribulose bisphosphate carboxylase small chain 1 [EC:4.1.1.39] type IAq	AFE_3052
*cbbQ2*	RubisCO activation protein CbbQ2	AFE_3053
*cbbO2*	RubisCO activation protein CbbO2	AFE_3054
***cbb3* operon**		
*hyp3*	16S RNA methyltransferase family	AFE_3255
*suhB*	Inositol-phosphate phosphatase	AFE_3254
*cbbF*	Fructose-1,6-biphosphatase [3.1.3.11]	AFE_3253
*cbbT*	Transketolase [2.2.1.1]	AFE_3252
*cbbG*	Glyceraldehyde-3-phosphate dehydrogenase type I [1.2.1.-]	AFE_3251
*cbbK*	Phosphoglycerate kinase [2.7.2.3]	AFE_3250
*pykA*	Pyruvate kinase II [2.7.1.40]	AFE_3249
*cbbA*	Fructose-biphosphate aldolase [4.1.2.13]	AFE_3248
*cbbE*	Ribulose-5-phosphate 3-epimerase [5.1.3.1]	AFE_3247
*cbbZ*	Phosphoglycolate phosphatase [3.1.3.18]	AFE_3246
*trpE*	Anthranilate synthase component I [4.1.3.27]	AFE_3245
*trpG*	Anthranilate synthase component II [4.1.3.27]	AFE_3244
*trpD*	Anthranilate phosphoribosyltransferase [2.4.2.18]	AFE_3243
*trpC*	Indole-3-glycerol phosphate synthase [4.1.1.48]	AFE_3242
***cbb4* operon**		
*metK*	*S*-adenosylmethionine synthase [2.5.1.6]	AFE_0532
*sahA*	*S*-adenosyl-L-homocysteine hydrolase [3.3.1.1]	AFE_0534
*metF*	5,10-methylenetetrahydrofolate reductase [1.7.99.5]	AFE_0535
*cbbP*	Phosphoribulokinase [2.7.1.19]	AFE_0536
*ynbD*	Single-stranded DNA specific exonuclease	AFE_0537
***cbb5* operon**		
*cbbM*	Ribulose bisphosphate carboxylase (RubisCO type II)	AFE_2155
*cbbQ*	RubisCO activation protein	AFE_2156
*cbbO*	RubisCO activation protein	AFE_2157
*cbbRm*	RubisCO operon transcription regulator	AFE_2158
***can* gene cluster**		
*can*2	Cytoplasmic carbonic anhydrase,β-type	AFE_0287
*sulP*	Predicted bicarbonate transporter	AFE_0286

*Predicted Enzyme Comission [EC] numbers are provided in the square brackets where available. ^*a*^Locus tags referenced in Esparza et al. (2010) have been discontinued and the most recent designations are provided here. Predicted Enzyme Commission [EC] numbers are provided in the square brackets.*

## Results and Discussion

### Growth of *A. ferrooxidans* in Varying CO_2_ Concentrations

**FIGURE 1:**
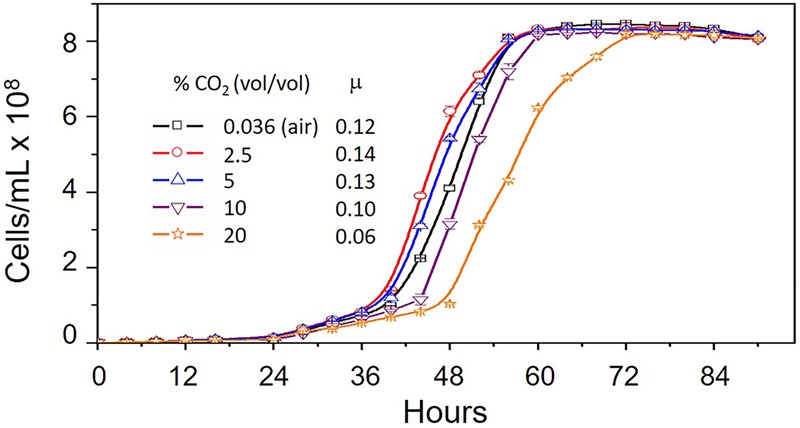
Growth curves of *Acidithiobacillus ferrooxidans* grown in 9K medium supplemented with 5 g/L elemental sulfur at 30°C in increasing concentrations of CO_2_ from 0.036% (vol/vol) (air) to 20% (vol/vol). Ranges are shown for duplicate measurements. μ = growth rate (hr^-1^).

In order to evaluate the effect of CO_2_ on the growth of *A. ferrooxidans*, cells were cultivated in 9K medium, pH 3.5 and containing 5 g/L elemental sulfur at 30°C (Quatrini et al., 2007) with increasing concentrations of CO_2_ from 0.036% (air) to 20%. Maximum growth rate occurred in 2.5% CO_2_ with decreasing growth rates in 5, 0.036, 10 and 20% CO_2_, respectively (Figure 1). However, maximum cell concentration (cells/mL) was unaffected by increasing CO_2_.

### Transcriptional Response of CBB Genes to Cellular Growth in Different CO_2_ Concentrations

**FIGURE 2:**
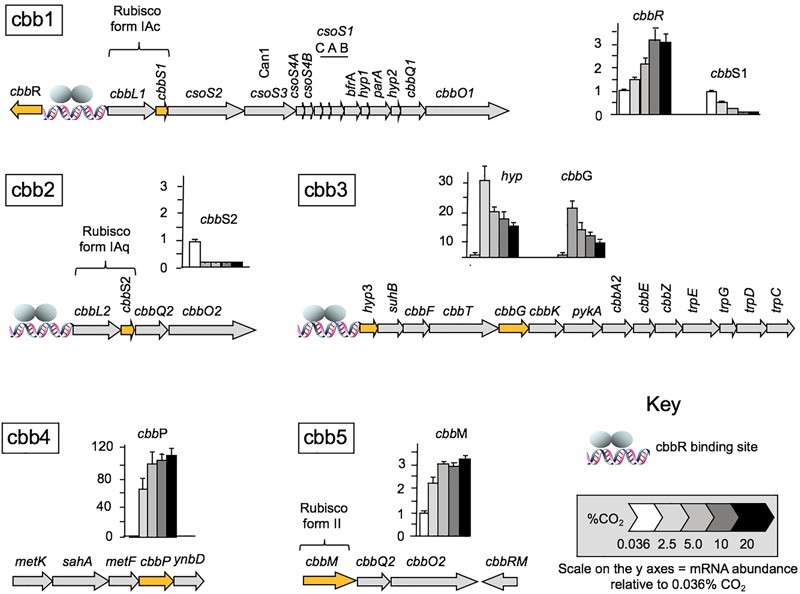
RNA transcript levels for genes and corresponding cbb operons involved in *A. ferrooxidans* CO_2_ assimilation under different CO_2_ concentrations relative to the transcript levels in air (0.036% CO_2_) normalized to one. mRNA abundance was determined by RT-qPCR (*n* = 4) for the following genes: *cbb*R encoding the transcriptional regulator CbbR (cbb1); *cbb*S1 small subunit of RubisCO Form 1Ac (cbb1), *cbb*S2 small subunit of RubisCO form 1Aq (cbb2); *hyp* (hypothetical) and *cbb*G glyceraldehyde-3-phosphate dehydrogenase (cbb3); *cbb*P phosphoribulokinase (cbb4) and *cbb*M RubisCO form II (cbb5). The genes assayed in each operon are highlighted in orange. The *cbbR* responsive promotors are indicated with a DNA symbol and a cartoon of the two subunits of CbbR1 (gray ellipses). A full list of genes in the operons is provide in Table 1.

Having established that CO_2_ concentration impacts cell growth rate, we wished to examine the effect of CO_2_ concentration on the expression of genes involved in the CBB and CCM pathways. Levels of RNA transcripts were assayed by RT-qPCR for one or more representative genes of each of the five cbb operons isolated from cells grown under different regimens of CO_2_ concentration from 0.036% (natural CO_2_ concentration in air) up to 20% (Figure 2). Transcript numbers of each tested gene are reported with respect to the level of RNA during growth at 0.036% CO_2_ normalized to one. The relative levels of transcripts of *cbbR* encoding the CbbR transcriptional regulator increased 3.4 ± 0.6-fold at a concentration of 10% CO_2_. A further increase to 20% CO_2_ did not result in any additional changes in RNA expression (Figure 2). In contrast, levels of RNA expression decreased with increasing CO_2_ concentrations for genes in the cbb1 operon including RubisCO Form IAc, associated carboxysome genes (including *can*1 encoding a carboxysome-associated 𝜀-type carbonic anhydrase), and the RubisCO activase genes *cbb*Q1 plus *cbb*O1. The expression of RNA from the cbb2 operon, encoding RubisCO Form IAq and the RubisCO activase genes *cbb*Q2 and *cbb*O2, also decreased with increasing CO_2_ but the decrease was more abrupt than that for RubisCO Form IAc suggesting that its expression was more sensitive to increasing CO_2_. RNA transcripts for *hyp*3 (unknown function) and *cbb*G (encoding glyceraldehyde-3-phosphate dehydrogenase) in the cbb3 operon were increased 30- and 20-fold, respectively when the CO_2_ concentration was raised from air to 2.5% CO_2_ followed by a subsequent decrease in transcript numbers in 5, 10, and 20% CO_2_, although transcripts in 20% CO_2_ were still higher than in air. The operon cbb3 encodes enzymes in the Calvin cycle together with phosphoglycolate phosphatase (*cbb*Z) that is involved in the detoxification of 2-phosphoglycolate produced by the reaction of RubisCO with oxygen (Ogren and Bowes, 1971) and part of the Trp operon that is involved in pyruvate formation and tryptophan biosynthesis. Transcripts for *cbb*P encoding phosphoribulokinase (PRK) in the cbb4 operon increased about 70-fold when cells were grown in 2.5% CO_2_ with a further increase in 5% CO_2_ to approximately 100-fold. Although the fold difference increased further in 10 and 20% CO_2_, the increases were not statistically significant using the Tukey test (Tukey, 1949) and ANOVA (Kotz et al., 2014). The *P*-value was 0.05. PRK catalyzes the ATP-dependent phosphorylation of ribulose 5-phosphate (RuP) into ribulose 1,5-bisphosphate (RuBP) which is the substrate for RubisCO.

RNA transcript abundance for *cbb*M (cbb operon 5), encoding RubisCO form II increased about two-fold in 2% CO_2_ with further increases to about threefold in 5–20% CO_2_.

### Protein Response of CBB Genes to Cellular Growth in Different CO_2_ Concentrations

**FIGURE 3:**
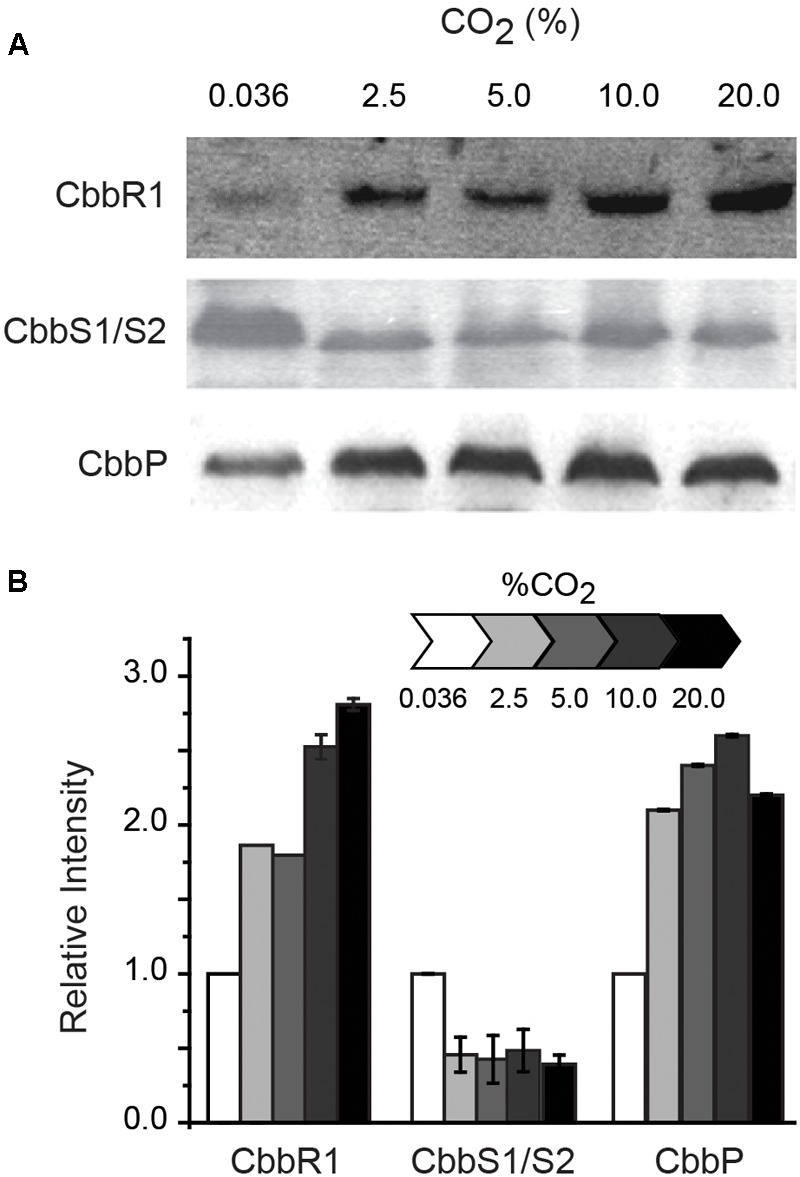
**(A)** Representative Western blots (of duplicates) of CbbR (cbb operon 1), CbbS1/S2 (the antibody used in the assay cannot distinguish between the two proteins) (cbb operon 1 and/or 2), and CbbP (cbb operon 4). **(B)** Quantification of the intensity of the Western blots relative to expression in 0.036% CO_2_ normalized to 1. Cells were grown in different concentrations of CO_2_ as indicated above the Western blots in **(A)** and in the scale bar in **(B)**. Error bars depict ranges of duplicate values.

RNA transcript abundance, as measured by RT-qPCR, does not always correspond to the level of the corresponding protein (Rocca et al., 2015). In order to evaluate whether protein concentration exhibited similar trends as the RNA levels, proteins encoded by selected cbb operon genes were assayed by Western blotting when cells were grown in increasing concentrations of CO_2_ (Figure 3). CbbR concentrations increased with increasing CO_2_ concentrations, mimicking transcript changes. The levels of CbbS1 and/or CbbS2 (the antibody cannot distinguish between the two forms of CbbS) decreased when cells were grown in increasing concentrations of CO_2_. Levels of CbbP increased until a concentration of 10% CO_2_ was reached with a subsequent slight decrease with 20% CO_2_. These data matched the changes in levels of RNA abundance in all three cases. However, absolute levels of protein abundance do not match transcript abundance, perhaps because of additional levels of post-transcriptional and post-translational regulation of the proteins and because Western-blotting is at best semi-quantitative (Gassmann et al., 2009).

### Ci Uptake

High affinity NDH-I_3_ and low affinity NDH-I_4_ CO_2_ uptake systems have been described in cyanobacteria (reviewed in Klanchui et al., 2017). However, bioinformatic examination of the genome of *A. ferrooxidans* failed to reveal gene candidates for the critical *cup*A in the NDH-I_3_ system or *cup*B in the NDH-I_4_ system, suggesting that *A. ferrooxidans* does not use these systems. Instead, we propose that CO_2_ passively diffuses into *A. ferrooxidans*, as has been shown in other organisms (Gutknecht et al., 1977).

### SulP Is Predicted to Encode Be a Bicarbonate Uptake/Efflux Pump

In order to investigate the possibility that SulP in *A. ferrooxidans* encodes a bicarbonate transporter, a detailed bioinformatic examination of the gene/protein was undertaken. SulP is predicted to be an inner membrane protein with eleven transmembrane regions with a similar topology to the experimentally verified BicA from *M. tuberculosis* H37Rv (Supplementary Figure S1). Particularly significant is that *sul*P is juxtaposed to *can*2 in *A. ferrooxidans*. *Can*2 is strongly predicted to encode a cytoplasmic carbonic anhydrase of the β-class clade B (Valdes et al., 2008). Carbonic anhydrases (EC 4.2.1.1) are metallo-anhydrases that catalyze the reversible hydration of CO_2_ to HCO_3_^-^ (Frost and McKenna, 2014). The juxtaposition of *sul*P and *can*2 suggests a functional relationship involving the uptake (or export) of HCO_3_^-^ by SulP and the interconversion of HCO_3_^-^ and CO_2_ by Can2 inside the cell. This hypothesis is strongly supported by the discovery, using the String database (Szklarczyk et al., 2017), of multiple examples of conserved microsynteny between *sul*P and *can* including gene fusions in many different organisms (Supplementary Figure S2).

**FIGURE 4:**
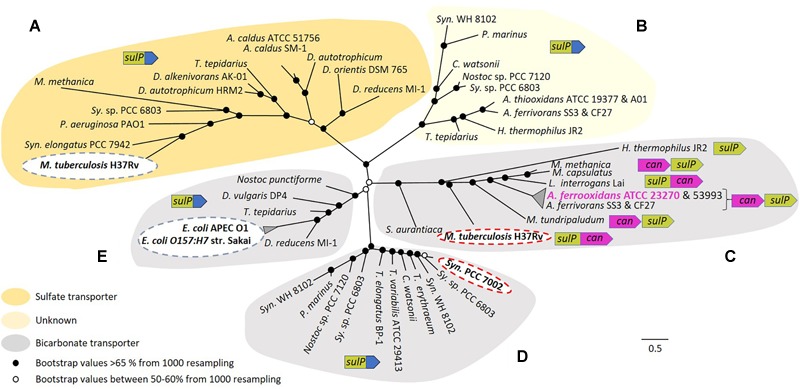
Maximum likelihood unrooted phylogenetic tree of SulP annotated as a sulfate (orange background), bicarbonate transporter (gray background) or of unknown function (light yellow background). **(A–E)** Refer to five phylogenetically distinct clades of sulfate or bicarbonate transporters. Bootstrap values between 50–60% (from 1000 resampling) are shown by white circles and bootstrap values >65% (1000 resampling) are represented by black circles. Arrows indicate genes where green = *sul*P, purple = *can* and blue = STAS domain. Abbreviations for species names: *A. caldus, Acidithiobacillus caldus; A. thiooxidans, Acidithiobacillus thiooxidans; A. ferrivorans, Acidithiobacillus ferrivorans; A. ferrooxidans, Acidithiobacillus ferrooxidans; C. watsonii, Crocosphaera watsonii*; *D. alkenivorans* AK-01, *Desulfatibacillum alkenivorans* AK-01; *D. autotrophicum* HRM2, *Desulfobacterium autotrophicum* HRM2; *D. orientis* DSM 765, *Desulfobacterium orientis* DSM 765; *D. reducens* MI-1, *Desulfobacterium reducens* MI-1; *D. vulgaris* DP4, *Desulfovibrio vulgaris* DP4; *E. coli* APEC O1, *Escherichia coli* APEC O1; *E. coli O157:H7* str. Sakai, *Escherichia coli O157:H7* str. Sakai; *H. thermophilus* JR2, *Hydrogenovibrio thermophilus* JR2; *L. interrogans* Lai, *Leptospira interrogans* Lai; *M. capsulatus, Methylococcus capsulatus*; *M. methanica, Methylomonas methanica*; *M. tuberculosis* H37Rv, *Mycobacterium tuberculosis* H37Rv; *M. tundripaludum, Methylobacter tundripaludum*; *Nostoc punctiforme, Nostoc punctiforme* PCC 73102; *P. aeruginosa* PAO1, *Pseudomonas aeruginosa* PAO1; *P. marinus, Prochlorococcus marinus; S. aurantiaca, Stigmatella aurantiaca; Sy.* sp. PCC 6803, *Synechocystis* sp. PCC 6803 substr. PCC-P; *Syn. elongatus* PCC 7942, *Synechococcus elongatus* PCC 7942; *Syn.* PCC 7002, *Synechococcus* sp. PCC 7002; *Syn.* WH 8102, *Synechococcus* sp. WH 8102; *T. elongatus* BP-1, *Thermosynechococcus elongatus* BP-1; *T. erythraeum, Trichodesmium erythraeum*; *T. tepidarius, Thermithiobacillus tepidarius* DSM 3134; *T. variabilis* ATCC 29413, *Trichormus variabilis* ATCC 29413. The scale bar represents the number of substitutions per site. (Figure with bootstrap values, gene names, accession numbers and references can be found in Supplementary Figure S3).

Motivated by the mounting evidence that SulP is a bicarbonate transporter, the functional relationship between SulP and experimentally validated sulfate or bicarbonate transporters was explored using phylogenomic approaches. SulP sequences chosen for comparison included an experimentally validated sulfate transporter from *M. tuberculosis* H37Rv and experimentally validated bicarbonate transporters from *E. coli* APEC O1, *E. coli O157:H7* str. Sakai, *M. tuberculosis* H37Rv, and *Synechococcus* sp. PCC 7002 as specified in the Section “Materials and Methods.” Additional SulP protein sequences with predicted sulfate or bicarbonate transport functions were obtained from multiple phylogenetically distinct Bacteria and added to the analysis. A multiple sequence alignment was constructed using MAFFT and ClustalW alignment program. Resultant alignments were used to construct a maximum likelihood, unrooted phylogenetic tree that was visualized and annotated in Figtree (Figure 4).

Five phylogenetically distinct clades were detected (labeled A to E in Figure 4). In clade A, sequences cluster with the experimentally verified sulfate transporter Rv1739c from *M. tuberculosis* H37Rv (Marietou et al., 2018). Clade B contains no sequences with experimentally validated functions and remains of unknown function. Clade C is associated with the experimentally validated bicarbonate transporter Rv3273 from *M. tuberculosis* H37Rv (Felce and Saier, 2004). SulP from *A. ferrooxidans* ATCC 23270 clusters in this clade, strongly supporting the contention that it is a bicarbonate and not a sulfate transporter. SulP sequences from *A. ferrooxidans* ATCC 53993 and *Acidithiobacillus ferrivorans* SS3 plus CF27 also cluster in this clade suggesting that they are also bicarbonate transporters. Microsynteny examination of clade C indicated that *sul*P is always juxtaposed to *can*2 and in some instances they are fused, providing additional support for the idea that the two genes are functionally related. Bicarbonate transporters in other systems use either Na^+^ or H^+^ as the counter-ion for the importation of HCO_3_^-^ (Saier et al., 2016). The counter-ion used by *A*. *ferrooxidans* remains unknown. Clade D includes sequences that cluster with the experimentally verified bicarbonate transporter BicA of *Synechococcus* PCC 7002 (Price et al., 2004). In clade E, sequences cluster with experimentally verified bicarbonate transporters YchM of *E. coli* APEC O1 and *E. coli O157:H7* str. Sakai (Moraes and Reithmeier, 2012). In contrast to clade C, SulP in all other clades is not associated with Can2 rather it is fused to a STAS domain (sulfate transporter/anti-sigma factor antagonist) that is thought to be involved in regulation or targeting (Shibagaki and Grossman, 2006).

### Transcriptional Response of the *can*2-*sul*P Gene Cluster to Cellular Growth in Different CO_2_ Concentrations

**FIGURE 5:**
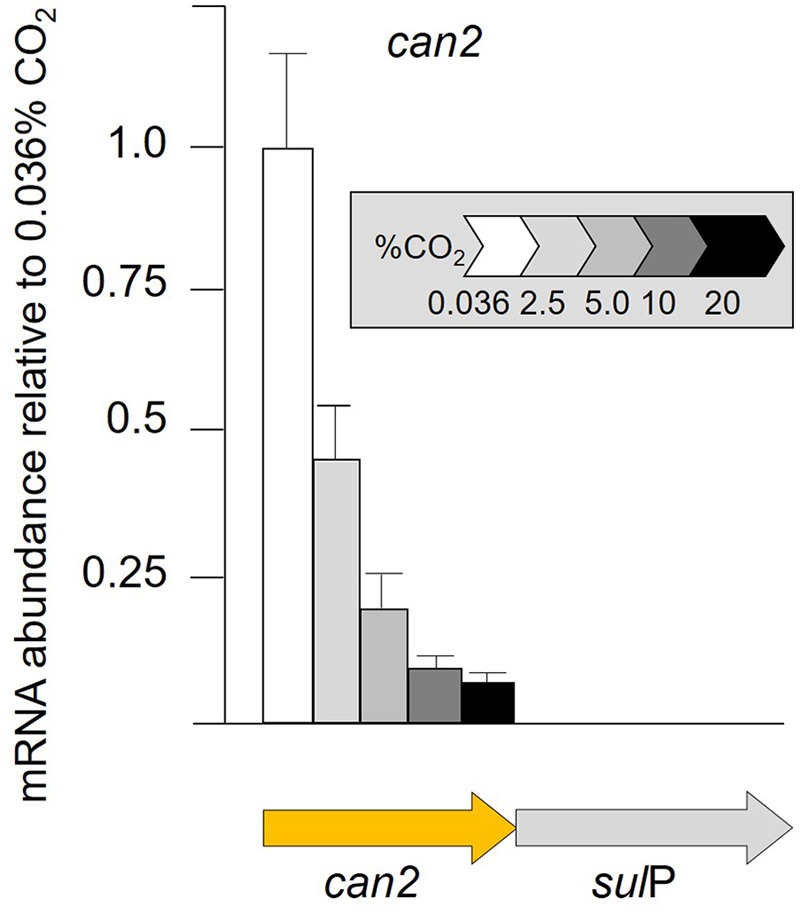
RNA transcript abundance (assayed by RT-qPCR) of *can*2 when cells were grown in different concentrations of CO_2_ from 0.036% CO_2_ (air) to 20% CO_2_. Orange arrow indicates the gene assayed for transcript abundance.

Given the multiple lines of bioinformatic evidence suggesting that *sul*P encodes a bicarbonate transporter and that it is functionally related to the adjacent *can*2 encoding carbonic anhydrase, transcript abundance of *can*2 was assayed by RT-qPCR when cells were grown in increasing concentrations of CO_2_ (Figure 5). RNA transcript abundance in 2% CO_2_ decreased to less than one-half that determined in 0.036% CO_2_, with further decreases to 0.1% in 20% CO_2_. The *can*2-*sul*P gene cluster has not been experimentally demonstrated to be an operon, but their phylogenetically conserved juxtaposition and close proximately separated by only nine nucleotides suggest that they are co-transcribed.

### Additional Discussion and Model

**FIGURE 6:**
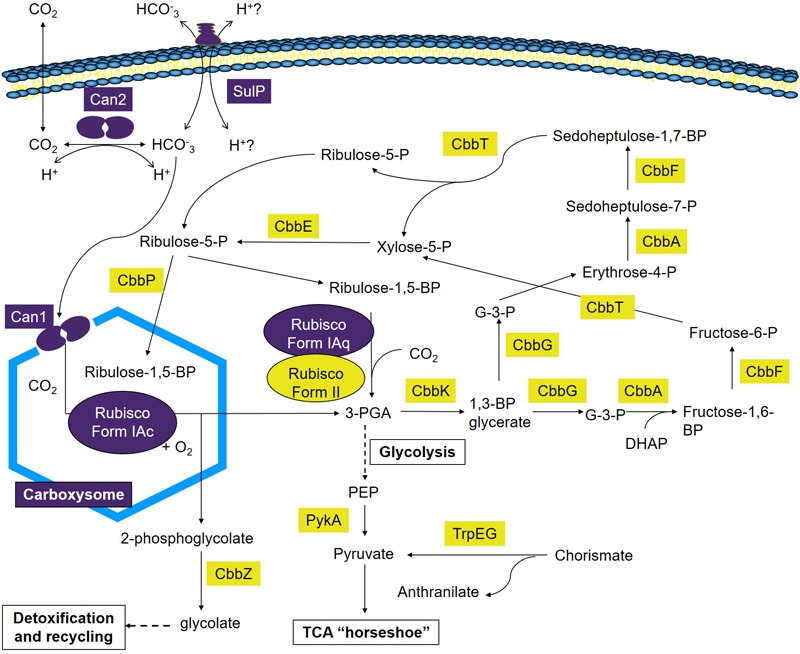
Model of Ci uptake and assimilation in *A. ferrooxidans*. Predicted genes and complexes involved in Ci uptake and assimilation are color coded where purple = genes and complexes with fewer RNA transcripts in cells grown at 2.5% CO_2_ versus air (O.036% CO_2_) and yellow = genes with an increase in transcript abundance in cells grown at 2.5% CO_2_ versus air (O.036% CO_2_). 1,3-BP glycerate, 1,3-bisphosphoglycerate; Fructose-1,6-BP, fructose-1,6-bisphosphate; Fructose-6-P, fructose-6-phosphate; G-3-P, glyceraldehyde-3-phosphate; 3-PGA, 3-phosphoglycerate; Ribulose-1,5-BP, ribulose-1,5-bisphosphate; Ribulose-5-P, ribulose-5-phosphate; Erythrose-4-P, Erythrose-4-phosphate; Sedoheptulose-7-P, sedoheptulose-7-phosphate; Sedoheptulose-1,7-BP, sedoheptulose-1,7-bisphosphate; Xylose-5-P, xylose-5-phosphate; PEP, phosphoenol pyruvate; DHAP, dihydroxyacetone phosphate.

This study advances our understanding of the mechanisms employed by *A. ferrooxidans* to take-up and concentrate Ci and the incorporation of CO_2_ into fixed carbon via the CBB cycle. A model is presented that builds upon prior investigations (Appia-Ayme et al., 2006; Esparza et al., 2009, 2010, 2015) and provides a preliminary framework to understand carbon fixation at extremely acidic pH under different regimes of CO_2_ concentration (Figure 6). Though much remains to validate aspects of the model, this work is an important step toward identifying the components, pathways, and regulation of carbon sequestration in *A. ferrooxidans*. It generates a more accurate and perceptive starting point to characterize the genetics and physiology of carbon sequestration in other extreme acidophiles. In addition, the model reveals a potentially flexible metabolic repertoire mediating carbon sequestration in different environments that can guide future research. Finally, it serves as a portal for deducing aspects of the CCM and CBB pathways in metagenomes from low pH environments (Guo et al., 2013).

### Model

The maximum rate of *A. ferrooxidans* growth in media containing elemental S as an energy source was obtained in the presence of 2.5% CO_2_ (Figure 1). This tendency can be explained, at least partially, by the expression of the five cbb operons as determined by changes in RNA transcript abundance (Figure 2) supported by protein abundance profiling (Figure 3). Two representative genes of the cbb3 operon, *hyp*3 and *cbb*G, obtain maximum transcript abundance in 2.5% CO_2_ that subsequently diminishes as the CO_2_ concentration is increased to 20% CO_2_. These genes are part of an operon coding for enzymes that pass the carbon from 3-PGA (3-phosphoglycerate), generated by RubisCO, through the pentose phosphate and glycolysis pathways to pyruvate and pathways for glycogen metabolism. The cbb3 operon also encodes *cbb*Z involved in 2-phosphoglycolate detoxification, a by-product of the reaction of RubisCO with O_2_ (Esparza et al., 2009, 2010, 2015). Assuming that a higher level of transcript abundance results in a concomitant increase in the levels of their respective encoded enzymes, growth in 2.5% CO_2_ could result in an increase in fixed carbon compounds and increased protection from O_2_ damage compared to growth in air. This, in turn, could contribute to more rapid growth in 2.5% CO_2_.

In order to achieve this increase in growth, the CBB cycle needs to provide more 3-PGA as a starting material to feed into the sugar transformation pathway. 3-PGA is the primary product of RubisCO and in 2.5% CO_2_ there is an increase in the abundance of transcripts for RubisCO form II encoded by *cbb*M of the cbb5 operon (Figure 2) that could account in part for an increase in 3-PGA production. In other organisms, Form II RubisCO has poor affinity for CO_2_ and a low discrimination against O_2_ as an alternative substrate suggesting that the enzyme is adapted to functioning in low-O_2_ and high-CO_2_ environments (Dobrinski et al., 2005; Badger and Bek, 2008). The observed increase in transcript abundance for RubisCO Form II in higher concentrations of CO_2_ (Figure 2) is consistent with this view.

One of the products of the enzymes encoded by the cbb3 operon is ribulose-5-P that is a precursor to ribulose-1,5-P, the substrate for RubisCO (Figure 6). The conversion of ribulose-5-P to ribulose-1,5-P is carried out by phosphoribulokinase (PRK) encoded by *cbb*P of the cbb4 operon. PRK catalyzes the ATP-dependent phosphorylation of ribulose 5-phosphate (RuP) into ribulose 1,5-bisphosphate (RuBP) that are both intermediates in the CBB Cycle. Together with RubisCO, PRK is unique to this cycle. There is a 65-fold increase in transcript abundance for *cbb*P in 2.5% CO_2_ compared to air (Figure 2) that could be responsible for an increase in ribulose-1,5-P. RNA transcript abundance for *cbb*P continues to rise in 10% CO_2_ but this is not accompanied by a concomitant increase in growth rate (Figure 1). Clearly, there are other factors limiting the growth rate at concentrations of CO_2_ above 2.5%. One possible explanation is the observed decrease in transcript abundance of genes in the cbb3 operon (encoding many genes involved in sugar interconversions) in 5–20% CO_2_ (Figure 2) that would potentially limit growth by diminished enzyme availability for various sugar conversions (Figure 6).

In summary, the model suggests *A. ferrooxidans* grows fastest in 2.5% CO_2_ due to an increase in transcript abundance for sugar transformation pathway genes, transcripts for *cbb*P that feeds RBP into the CBB cycle, and transcripts for genes encoding RubisCO Form II that is postulated to be the RubisCO used at higher CO_2_ concentrations.

### Carbon Concentration Mechanisms: Carboxysomes With β-Type Carbonic Anhydrases

Another important consideration is how *A. ferrooxidans* CCM genes involved in the uptake and concentration of Ci respond to changes in CO_2_ concentration. *A. ferrooxidans* has evolved efficient CCMs to transport and accumulate Ci. First, it encodes the formation of α-carboxysomes, bacterial micro-compartments that provide elevated concentrations of CO_2_ to the main CO_2_-fixing enzyme RubisCO and reduce its reaction with oxygen (reviewed in Rae et al., 2013). *A. ferrooxidans* has multiple forms of RubisCO including two copies of Form I and one copy of Form II (Heinhorst et al., 2002; Levican et al., 2008). Using protein similarity analysis, we now predict the two forms of Form I RubisCO as sub-types IAc and IAq that consist of the large and small subunits of RubisCO as has been observed in other organisms (Badger and Bek, 2008). RubisCO Form II consists only of a large subunit with little sequence or structural similarity with the large subunit of forms IAc and IAq (Tabita et al., 2008; Bohnke and Perner, 2017). Genes encoding RubisCO Form IAc are encoded in the cbb1 operon, co-occur with carboxysome formation genes, and is probably encapsulated within the carboxysome as has been found in other organisms (Tabita et al., 2008). In addition, *csoS3* is also present in the cbb1 operon and encodes a β-type carbonic anhydrase (Can1) (Sawaya et al., 2006). CsoS3 is located in the carboxysome shell and is responsible for the conversion of bicarbonate to CO_2_ and is an important contributor to CCM (So et al., 2004). Thus, cbb1 encodes the major components of the CCM carboxysome that encapsulates RubisCO in *A. ferrooxidans*.

Under conditions of low CO_2_ concentrations, carboxysome formation genes are upregulated in other microorganisms (Orus et al., 2001). This has been confirmed in *A. ferrooxidans* where increased transcript abundance was observed for cbb1 operon genes under anaerobic conditions with low CO_2_ concentrations (Osorio et al., 2013). On the other hand, genes encoding RubisCO Form IAq, located in the cbb2 operon, are not closely linked in the genome to carboxysome genes. In addition, despite considerable sequence similarity of both large and small subunits of Form IAq and Form IAc, a major difference is the presence in many bacteria including *A. ferrooxidans* of a six amino acid insertion in the small subunit of Form IAq not found in Form IAc (Supplementary Figure S4). A crystal structure of the small subunit of Form IAc RubisCO from *Halothiobacillus neapolitanus* [1SVD; the structure of *H. neapolitanus* RubisCO, (Kerfield, C. A. et al., 2005, unpublished)] provides evidence that this insertion impedes its interaction with carboxysome proteins (Badger and Bek, 2008), suggesting that Form IAq is not associated with carboxysomes in the cell. An examination of its kinetic properties suggests that Form IAq is adapted to environments with medium to high CO_2_ concentrations with oxygen present whereas Form IAc is more adapted to low CO_2_ and low to high O_2_ environments (Badger and Bek, 2008). RNA transcript abundance of both RubisCO Form IAc and Form IAq indicate that their abundance diminishes in CO_2_ concentrations above that of air. However, as both their expressions are low it is not possible to discern if there are statistically significant differences in the rate of decrease of transcript abundance between the two RubisCO Forms as CO_2_ concentrations are increased.

In summary, the *A. ferrooxidans* genome encodes α-carboxysomes that include β-type carbonic anhydrases and a type IAc RubisCO. The genes for these functions are present in operon cbb1 under the control of CbbR. RNA transcript abundance for cbb1 decreases with increasing CO_2_ concentrations. It is most likely that the carboxysomes and associated functions are used as a CCM mechanism in low CO_2_ concentrations. In contrast, RubisCO Type IAq (cbb2 operon) and RubisCO Type II (cbb5 operon) are probably not encapsulated in carboxysomes. RNA transcript abundance for RubisCO Type II increases in higher CO_2_ concentrations and may be the principle RubisCO used at higher concentrations of CO_2_ (or lower O_2_ concentrations). The role of RubisCO Type IAq is not clear but may represent a form that is used at slightly higher concentrations of CO_2_ or during rapid fluxes of CO_2_ concentration.

### Carbon Concentration Mechanisms: Bicarbonate Uptake and Cytoplasmic β-Carbonic Anhydrase

In addition to the proposed involvement of a carboxysome β-type carbonic anhydrase, a second potential CCM mechanism is the presence of a cytoplasmic-located β-carbonic anhydrase (Can2) that is genetically linked to a predicted bicarbonate transporter SulP. Carbonic anhydrases catalyze the proton-mediated reversible hydration of CO_2_ to HCO_3_^-^ (Smith and Ferry, 2000) equilibrating the reaction between CO_2_, bicarbonate, and protons and play important roles in ion transport, acid-base regulation, gas exchange, and CO_2_ fixation in many organisms (Lotlikar et al., 2013; Aggarwal and McKenna, 2015). Although the function of Can2 in *A. ferrooxidans* remains to be experimentally validated, the model suggests that it is involved in the reversible hydration of CO_2_ (that has entered the cell by diffusion) to HCO_3_^-^ as found in other organisms (Smith and Ferry, 2000). The genomic co-localized of *can*2 with the predicted bicarbonate transporter *sul*P suggests that they work together, perhaps in pH regulation as has been found in other organisms (Lotlikar et al., 2013; Aggarwal and McKenna, 2015). In this model, the importation of bicarbonate into the cell by SulP would be accompanied by the expulsion of protons. Subsequently, Can2 could convert the bicarbonate to CO_2_ accompanied by the conversion of cellular protons to water and the diffusion of the CO_2_ outside the cell as shown in Figure 6. Thus, protons are both exported and consumed and this may be an important mechanism for pH regulation in extremely acidic conditions. Alternatively, and not mutually exclusive, there is the possibility that Can2 works in the reverse direction and converts CO_2_ to bicarbonate that is subsequently taken into the carboxysome by Can1. This could improve the efficiency of carbon fixation under limiting conditions of external CO_2_ as has been observed in the facilitation of growth of other microbes at low partial pressures of CO_2_ (Kusian et al., 2002; Merlin et al., 2003; Mitsuhashi et al., 2004; Burghout et al., 2010). Low partial pressures of CO_2_ in bioleaching heaps has been observed due to the decreased solubility of CO_2_ in low pH especially when the temperature of the heap rises (lowering further the solubility of CO_2_), resulting from chemical and biochemical exothermic reactions including the conversion of pyrite to oxidized sulfur compounds (Valdés et al., 2010). If Can2 is involved in improving uptake of CO_2_ at low partial pressures of CO_2_, then it might explain why *can*2 exhibits a decrease in transcript abundance in increasing concentrations of CO_2_ (Figure 2).

In summary, *A. ferrooxidans* is predicted to have a second carbonic anhydrase, encoded by *can*2, located in the cytoplasm that functions in the reversible hydration of CO_2_. Juxtaposed is a gene (*sul*P) predicted to be membrane associated bicarbonate transporter. It is predicted that *sul*P/*can*2 constitute an operon. The abundance of transcripts for *sul*P/*can*2 decreases with increasing CO_2_ concentrations. It is hypothesized that SulP/Can2 function as a bicarbonate uptake system but they may also serve as an intracellular proton concentration homeostatic mechanism.

### Regulation

Regulation of Ci uptake and assimilation is very complex and is dependent on transcriptional regulators that act in concert with small molecular effectors that are well known metabolites. In addition, it has recently been discovered that numerous small RNA molecules act as antisense regulators (Burnap et al., 2015). Although there are many studies of the regulation of Ci uptake and assimilation in autotrophs, principally in photoautotrophs (Kusian and Bowien, 1997), there have been only limited insights into their regulation in extremely acidophilic chemolithoautotrophs. It was suggested in Esparza et al. (2010) that the regulation of cbb operons 1–4 of *A. ferrooxidans* involved the action of the master regulator CbbR, as has been observed in many microorganisms (Badger and Bek, 2008). The evidence included: (i) the presence of a CbbR binding site upstream of *cbb*R leading to autoregulation of *cbb*R (Esparza et al., 2015); (ii) the presence of CbbR binding sites upstream of operons cbb1-3 (Esparza et al., 2010); and (iii) the activity of *A. ferrooxidans cbb*R promoters when cloned into the surrogate host *C. necator* (formerly *R. eutropha*) (Esparza et al., 2015), including the detection of promoter activity upstream of cbb4 even in the absence of an experimentally validated CbbR binding site in this operon. The observed transcript profiles of operons cbb1-4 can be explained on the basis of the activity of CbbR. Increased CbbR down-regulates the expression of the cbb2 operon and up-regulates the cbb3 and cbb4 operons (Figure 4). That CbbR can act as both a positive and negative regulator has been observed in other organisms (Viale et al., 1991). However, what controls the up-regulation of CbbR in *A. ferrooxidans* in response to increasing CO_2_ concentrations is unknown. One possibility is that it involves the interaction of the regulator RegA, that responds to the redox state of the cell, with CbbR (Dangel and Tabita, 2015). Alternatively, it could involve the binding of possible effectors such as ATP, NADPH, RuBP, and fructose-1,6-bisphosphate to CbbR, many of which are metabolites of the Cbb cycle involved in feedback regulation (Joshi et al., 2012) as discussed further below. An important observation is the increase in transcripts of RubisCO Form II in high CO_2_ concentrations (Figure 2). In other organisms it has been shown that RubisCO Form II is controlled by the transcriptional regulator CbbRm (Bohnke and Perner, 2017) and CbbRm plus RubisCO Form II expression levels increase at CO_2_ concentrations above 2%. The molecular mechanisms underlying the regulation of RubisCO Form II are only beginning to be understood (Dubbs et al., 2004; Toyoda et al., 2005; Tsai et al., 2015). It has been suggested that RubisCO Form II evolved at least 2.7 billion years ago, when atmospheric CO_2_ levels were one to three orders of magnitude higher than today (Raven, 1991; Rye et al., 1995; Tortell, 2000; Kaufman and Xiao, 2003; Dobrinski et al., 2005; Griffiths et al., 2017). At that time, CCMs were perhaps not required and that is consistent with the observation that RubisCO Form II in *A. ferrooxidans* is not associated with the CCM carboxysome formation genes.

Regulation of expression of the CCM and CBB cycle genes in other organisms is also known to be mediated by small effector molecules (Dubbs et al., 2004; Tamoi et al., 2005). These include CO_2_ (Shimizu et al., 2015), α-ketoglutarate and the oxidized form of nicotinamide adenine dinucleotide phosphate (NADP^+^) (Daley et al., 2012), ATP, fructose 1,6-bisphosphate, and NADPH (Joshi et al., 2012) and several compounds of the CBB reductive pentose phosphate pathway several of which are encoded by the operon cbb3 of *A. ferrooxidans* (Figure 3, 6) (Dubbs et al., 2004). The role of these effectors has not been tested in *A. ferrooxidans* and it will be a considerable challenge to elucidate the manifold dependencies and interconnections between the diverse cellular processes that together facilitate the regulation of the CCM and CBB pathways in this organism. The use of the surrogate host *C. necator* provides an opportunity to experimentally test the role of metabolic effectors in *A. ferrooxidans* (Esparza et al., 2015).

In summary, CbbR has been shown to regulate the expression of cbb operons 1-4. Its increase in expression in higher CO_2_ concentrations is consistent with previous observations that it can serve as both a negative regulator (cbb operons 1 and 2) and a positive regulator (cbb4 operon). In the case of operon cbb3, an initial increase in expression is observed when the CO_2_ concentration is increased to 2.5% suggesting that CbbR acts as a positive regulator, but this is followed by subsequent decreases in transcript abundance as CO_2_ levels are increased beyond 2.5% indicating that other factors are involved in the regulation of cbb3. These factors are unknown but could include interactions with small metabolites and with the redox sensing RegAB system.

### High Level Network Interconnections

Network analyses of the multiple levels of CCM and CBB regulation including the regulation of bicarbonate uptake by a CbbR-like transcription factor (Omata et al., 2001), the interconnection between carbon and nitrogen metabolism (Wheatley et al., 2016) and with oxidative stress as sensed by the redox-sensitive two-component global regulator system RegAB (Romagnoli and Tabita, 2009), and other multilayered connections (Eisenhut et al., 2007; McClure et al., 2016; Westermark and Steuer, 2016) have been carried out principally in photoautotrophs. Less is known about the potential high level regulatory networks involved in Ci uptake and assimilation in extremely acidic chemolithoautotrophs7 (Campodonico et al., 2016). Of particular relevance to the present study, was the discovery that transcripts were more abundant for the glycogen biosynthetic pathway genes (*gly*B, EC 2.4.18; *glyC*, EC 2.7.7.27; *amy* and *ma*lQ, EC 2.4.1.25) when *A. ferrooxidans* was cultivated in sulfur versus ferrous iron and that this coincided with increased expression of CBB genes (Appia-Ayme et al., 2006). Glycogen biosynthesis/degradation has been shown to be interconnected with glycolysis and the pentose phosphate pathway in *A. ferrooxidans* (Mamani et al., 2016), supporting the idea that there is a direct connection with CBB cycle genes and the biosynthesis of glycogen. It is postulated that more energy is available when sulfur is used as an energy source and we propose that this is used as an opportunity to synthesize glycogen as a stored energy source as has been proposed in other organisms (Goh and Klaenhammer, 2013; Preiss, 2014).

### Ecological Considerations

The availability of Ci depends, in part, on the pH of the environment. At high pH values (>pH 9) it occurs principally as carbonate/bicarbonate (HCO_3_^-^/CO_3_^2-^). At circumneutral pH values, it is mainly available as bicarbonate, whereas in very low pH environments (<pH4) Ci occurs principally as a dissolved hydrated CO_2_ gas (H_2_CO_3_). In addition, to the different chemical forms of Ci, their concentrations can vary over a wide range in different environments (Sandrini et al., 2014, 2015; Klanchui et al., 2017).

Whereas Ci uptake has been studied extensively in cyanobacteria, to the best of our knowledge, there are no studies on the uptake of bicarbonate in very low pH environments. In initial studies using BlastP with an acceptance cut-off of 1e-06 to probe the genome of *A. ferrooxidans*, we were unable to detect any of the known bicarbonate transporters. However, weak sequence similarity of SulP with the bicarbonate transporter BicA was observed and additional phylogenomic and gene microsynteny studies supported the prediction that SulP was a bicarbonate transporter rather than the original prediction that it was a sulfate transporter (Figure 4). *A. ferrooxidans* grows optimally at pH 2.4 when ferrous iron is used as an energy source and would be expected to rely principally on the free diffusion of the hydrated CO_2_ gas (H_2_CO_3_) through the membrane as their source of Ci. So why does *A. ferrooxidans* have a predicted bicarbonate transporter?

Although *A. ferrooxidans* grows at a pH optimum of 2.5 when grown on ferrous iron medium, it can also grow at pH 5 when elemental sulfur is used as an energy source (Mcgoran et al., 1969). At this pH, and in an environment with a temperature of 25°C and a salinity of 5,000 ppm, up to 10% of the dissolved Ci could be in the form of bicarbonate (WikiVividly, 2018) and having a bicarbonate transporter would allow *A. ferrooxidans* to use this source of Ci. This would permit *A. ferrooxidans* to exploit a wide range of HCO_3_^-^ availability, providing potential access to environments with a spectrum of pH values from, e.g., pH 1 to at least pH 5. Thus *A. ferrooxidans* would be considered a “generalist” rather than a “specialist” (Baronchelli et al., 2013) in a dynamic environment such as a bioleaching heap where initial pHs are around 5–6 at a time when acid addition is consumed by, e.g., silica minerals (Dopson et al., 2008, 2009), and before sulfur compound oxidation to sulfuric acid has lowered the pH to the *A. ferrooxidans* optimum.

## Data Availability

The datasets generated for this study can be found in GenBank NCBI, NC_011761.

## Author Contributions

DH and EJ conceived the project. ME, EJ, and DH planned the experiments. ME carried out the experiments and CG helped with the bioinformatic analyses. All authors interpreted the results. DH and MD wrote the initial draft of the paper. All authors contributed to manuscript revision and approved the submitted version.

## Conflict of Interest Statement

The authors declare that the research was conducted in the absence of any commercial or financial relationships that could be construed as a potential conflict of interest.

## Footnotes

1 http://tree.bio.ed.ac.uk/software/figtree/

## Abbreviations

AMD: acid mine drainage
CBB: Calvin–Benson–Bassham
CCM: carbon concentration mechanism
Ci: inorganic carbon
