# Supplementary material for: Effect of CO2 Concentration on Uptake and Assimilation of Inorganic Carbon in the Extreme Acidophile Acidithiobacillus ferrooxidans
Source: Front Microbiol. 2019 Apr 4;10:603. doi: 10.3389/fmicb.2019.00603 (PMC6458275; doi:10.3389/fmicb.2019.00603)
Supplement: Supplementary file 1 [file Data_Sheet_1.PDF]

Effect of CO_2 Concentration on Uptake and Assimilation of Inorganic Carbon in the Extreme Acidophile *Acidithiobacillus ferrooxidans*

Mario Esparza, Eugenia Jedlicki, Carolina González, Mark Dopson, and David Holmes

SUPPLEMENTAL FIGURE S_1

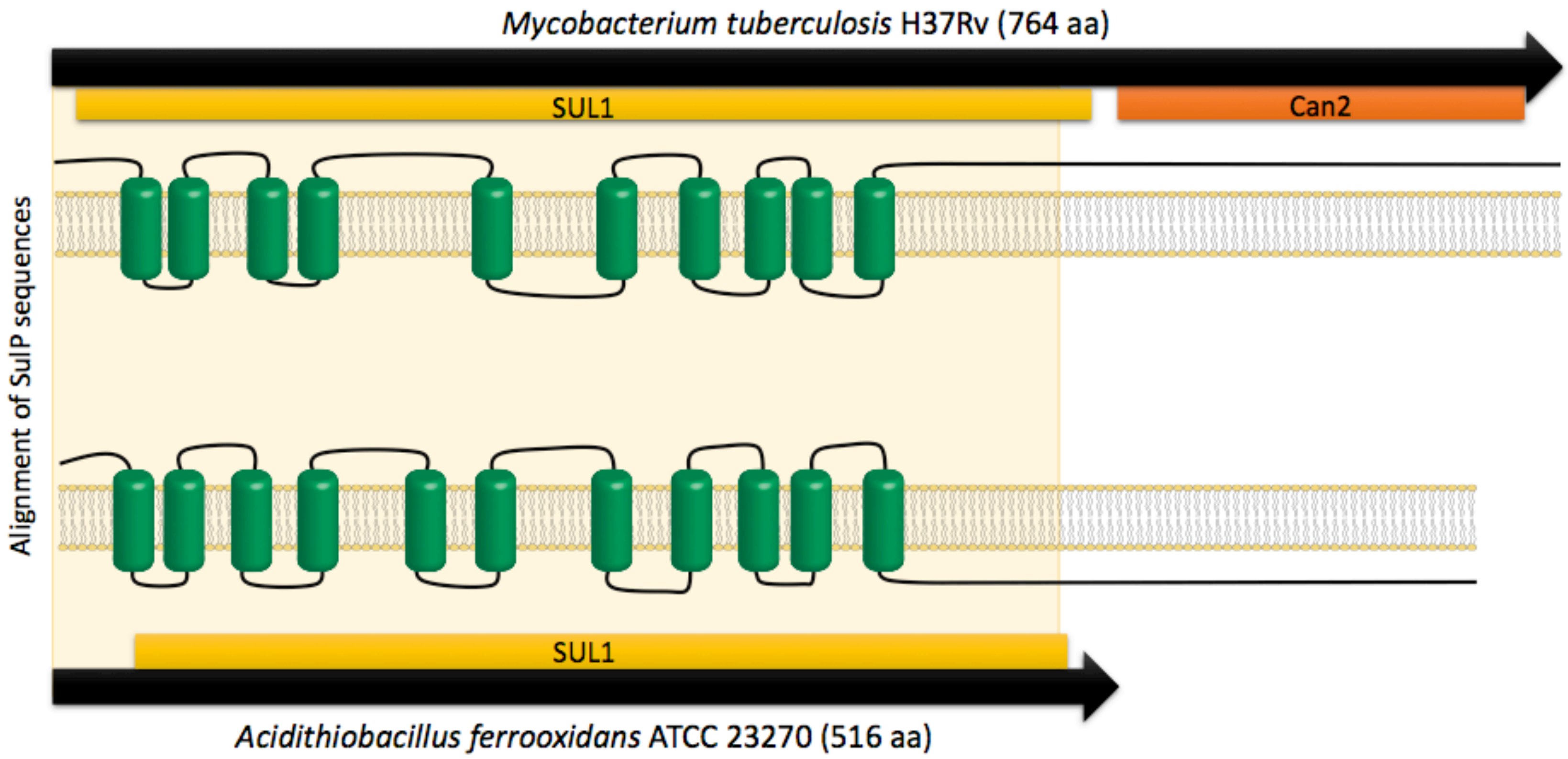

Alignments of the SulP-Can gene fusion of *Mycobacterium tuberculosis* H37Rv (NP_217790.1) with SulP of *Acidithiobacillus ferrooxidans* (ACK80903.1, locus tag AFE_0286). Black arrows represent the coding sequence of each gene. Above the black arrows are shown the domain structures, where Sul1 = COG0659: Sulfate permease or related transporter, MFS superfamily [Inorganic ion transport and metabolism] and CynT = COG0288: a member of the superfamily cl00391, Carbonic anhydrase [Inorganic ion transport and metabolism]. Above the domain structures, the predicted respective transmembrane domains are shown using the Phobius prediction server (Lukas Käll, Anders Krogh and Erik L. L. Sonnhammer. Advantages of combined transmembrane topology and signal peptide prediction – the Phobius web server. *Nucleic Acids Res.*, 35:W429-32, July 2007).
