# Supplementary material for: Effect of CO2 Concentration on Uptake and Assimilation of Inorganic Carbon in the Extreme Acidophile Acidithiobacillus ferrooxidans
Source: Front Microbiol. 2019 Apr 4;10:603. doi: 10.3389/fmicb.2019.00603 (PMC6458275; doi:10.3389/fmicb.2019.00603)
Supplement: Supplementary file 2 [file Data_Sheet_2.PDF]

# Effect of $CO_2$ Concentration on Uptake and Assimilation of Inorganic Carbon in the Extreme Acidophile *Acidithiobacillus ferrooxidans*

Mario Esparza, Eugenia Jedlicki, Carolina González, Mark Dopson, and David Holmes

## SUPPLEMENTAL FIGURE $S_2$

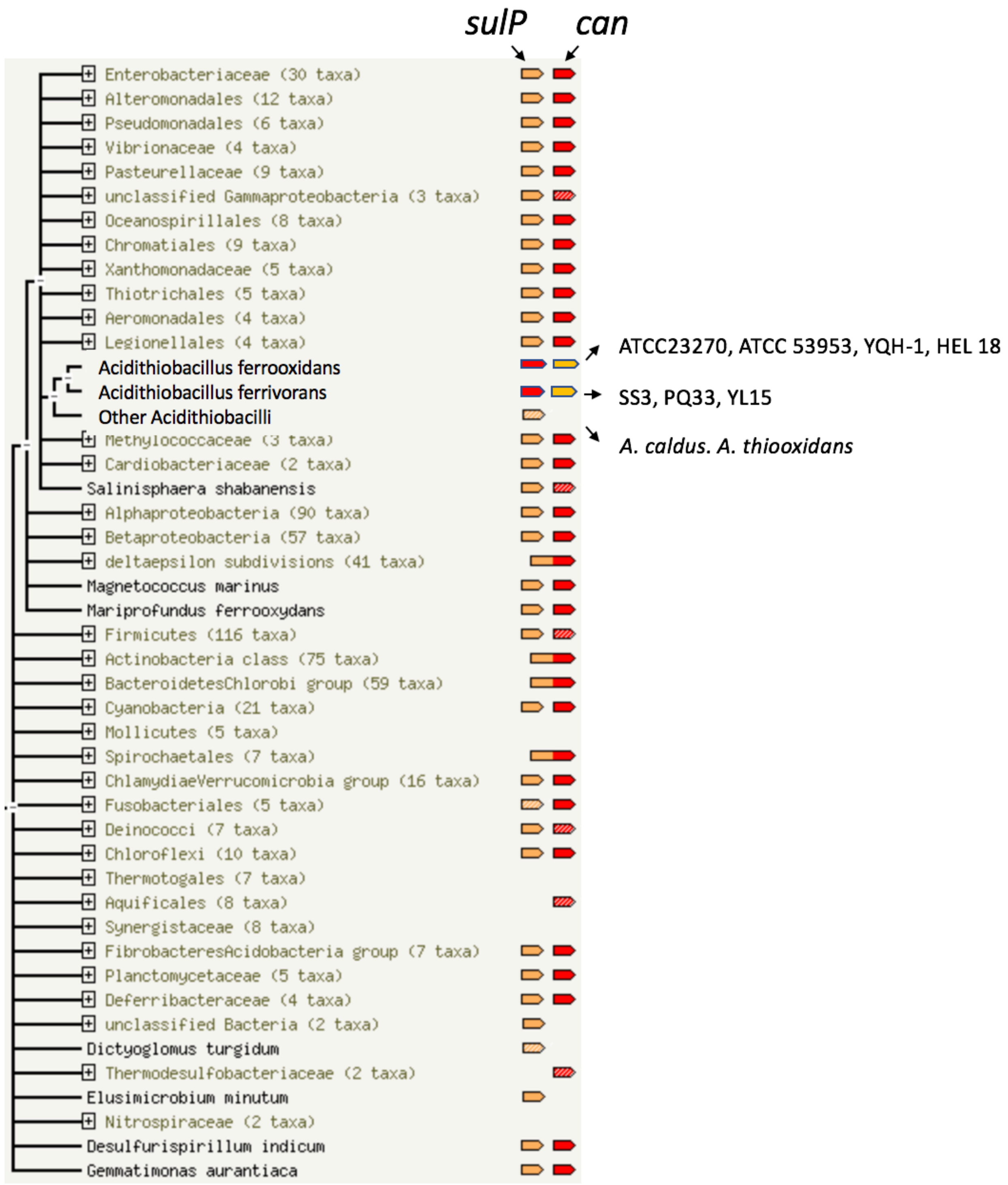


Cladogram of bacteria illustrating the distribution of gene neighborhoods and gene fusions of *sulP* and *can*, predicted to encode an inorganic ion transporter and a carbonic anhydrase, respectively. In most bacteria, the order of the genes is *SulP*- *can,* but in *A. ferrooxidans* and *A. ferrivorans* the order is *can-sulP*. There are many instances in which *sulP-can* are fused in one gene, strongly supporting the suggestion that they are functionally related. The cladogram was developed using STRING (string-db.org). The order of the genes in *A. ferrooxidans* and *A. ferrivorans* was incorrectly displayed in the original STRING output. This has been corrected in the above figure.
