# Supplementary material for: Effect of CO2 Concentration on Uptake and Assimilation of Inorganic Carbon in the Extreme Acidophile Acidithiobacillus ferrooxidans
Source: Front Microbiol. 2019 Apr 4;10:603. doi: 10.3389/fmicb.2019.00603 (PMC6458275; doi:10.3389/fmicb.2019.00603)
Supplement: Supplementary file 3 [file Data_Sheet_3.pdf]

Supplementary Figure S3

Effect of CO_2 Concentration on Uptake and Assimilation of Inorganic Carbon in the Extreme Acidophile *Acidithiobacillus ferrooxidans*

Mario Esparza, Eugenia Jedlicki, Carolina González, Mark Dopson, and David S. Holmes

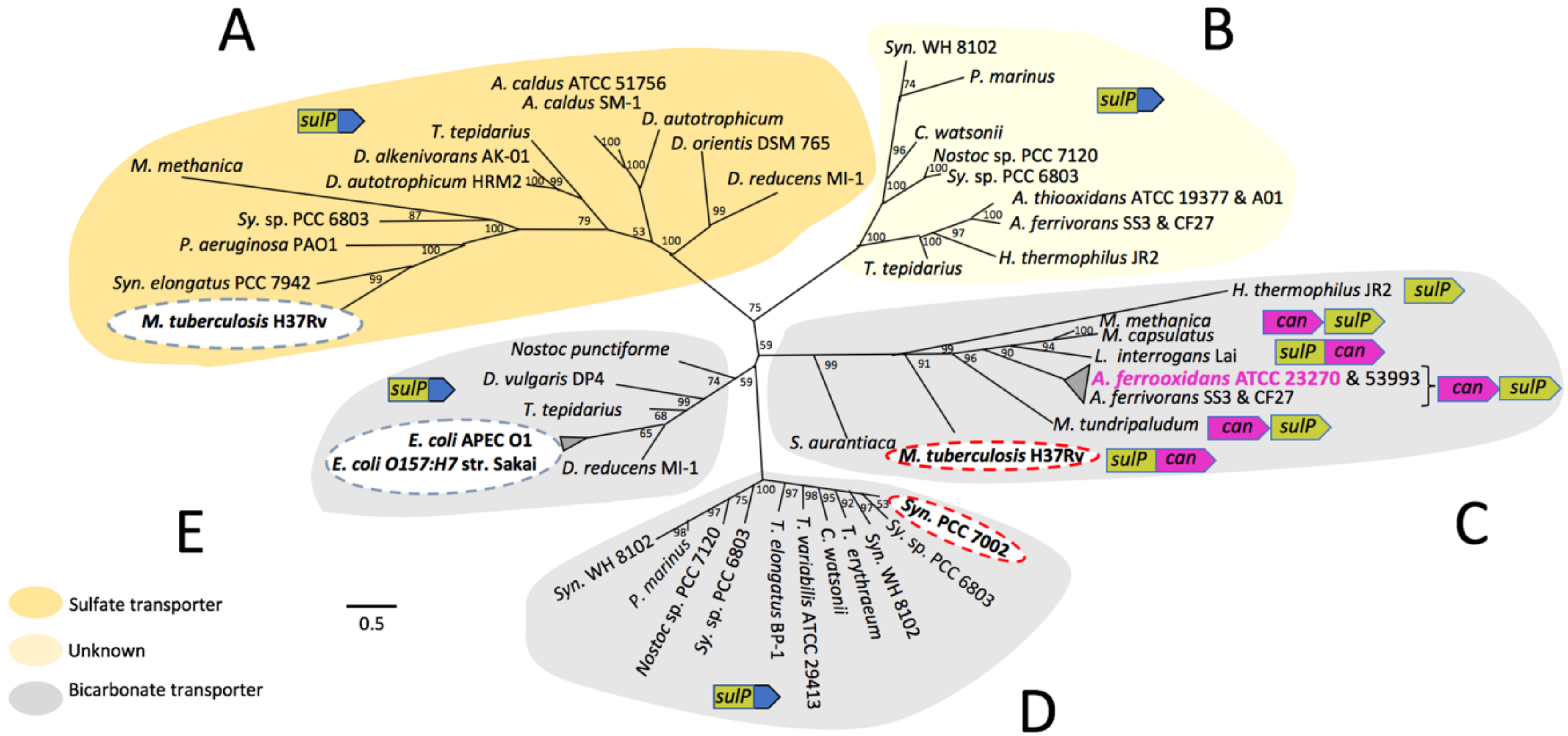

Figure 4. Maximum likelihood unrooted phylogenetic tree with bootstrap values of SulP annotated as a sulfate (orange background), bicarbonate transporter (grey background) or of unknown function (light yellow background). Labels A to E refer to five phylogenetically distinct clades of sulfate or bicarbonate transporters. Arrows indicate genes where green = sulP, purple = can and blue = STAS domain. Abbreviations for species names: *A. caldus*, *Acidithiobacillus caldus*; *A. thiooxidans*, *Acidithiobacillus thiooxidans*; *A. ferrivorans*, *Acidithiobacillus ferrivorans*; *A. ferroooxidans*, *Acidithiobacillus ferrooxidans*; *C. watsonii*, *Crocosphaera watsonii*; *D. alkenivorans* AK-01, *Desulfatibacillum alkenivorans* AK-01; *D. autotrophicum* HRM2, *Desulfobacterium autotrophicum* HRM2; *D. orientis* DSM 765, *Desulfobacterium orientis* DSM 765; *D. reducens* MI-1, *Desulfobacterium reducens* MI-1; *D. vulgaris* DP4, *Desulfovibrio vulgaris* DP4; *E. coli* APEC O1, *Escherichia coli* APEC O1; *E. coli* O157:H7 str. Sakai, *Escherichia coli* O157:H7 str. Sakai; *H. thermophilus* JR2, *Hydrogenovibrio thermophilus* JR2; *L. interrogans* Lai, *Leptospira interrogans* Lai; *M. capsulatus*, *Methylococcus capsulatus*; *M. methanica*, *Methylomonas methanica*; *M. tuberculosis* H37Rv, *Mycobacterium tuberculosis* H37Rv; *M. tundripaludum*, *Methylobacter tundripaludum*; *Nostoc punctiforme*, *Nostoc punctiforme* PCC 73102; *P. aeruginosa* PAO1, *Pseudomonas aeruginosa* PAO1; *P. marinus*, *Prochlorococcus marinus*; *S. aurantiaca*, *Stigmatella aurantiaca*; *Sy.* sp. PCC 6803, *Synechocystis* sp. PCC 6803 substr. PCC-P; *Syn. elongatus* PCC 7942, *Synechococcus elongatus* PCC 7942; *Syn.* PCC 7002, *Synechococcus* sp. PCC 7002; *Syn.* WH 8102, *Synechococcus* sp. WH 8102; *T. elongatus* BP-1, *Thermosynechococcus elongatus* BP-1; *T. erythraeum*, *Trichodesmium erythraeum*; *T. tepidarius*, *Thermithiobacillus* tepidarius DSM 3134; *T. variabilis* ATCC 29413, *Trichormus variabilis* ATCC 29413. The scale bar represents the number of substitutions per site.

Gene names, accession number, organisms and references for sulfate transporter (*sul*P-type) and bicarbonate transporters (*bic*A-type) used in this study to construct the phylogenetic tree shown in Figure 4 and Supp. Fig S_3.

Gene name	Accession	Organisms	Reference
**Group A**			
Sulfate ABC transporter permease	NP_216255.1	*Mycobacterium tuberculosis* H37Rv	Marietou et al. 2018
Similar to plant sulfate transporter	AAB88215.1	*Synechococcus elongatus* PCC 7942	Price et al. 2004
Sulfate transporter	NP_250338.1	*Pseudomonas aeruginosa* PAO1	Marietou et al. 2018
High affinity sulfate transporter	BAL34295.1	*Synechocystis* sp. PCC 6803 substr. PCC-P	Price et al. 2004
SulP family inorganic anion transporter	WP_013820177.1	*Methylomonas methanica*	Boden et al. 2011
SulP3	ACN17094.1	*Desulfobacterium autotrophicum* HRM2	Marietou et al. 2018
Sulfate permease	ACL04621.1	*Desulfatibacillum alkenivorans* AK-01	Marietou et al. 2018
STAS domain-containing protein	WP_028988753.1	*Thermithiobacillus tepidarius*	This study
SulP family inorganic anion transporter	WP_081662774.1	*Thermithiobacillus tepidarius*	This study
Sulfate permease	AIA56134	*Acidithiobacillus caldus* ATCC 51756	This study
sulfate transporter	AEK59090.1	*Acidithiobacillus caldus* SM-1	This study

SulP2	ACN16424.1	*Desulfobacterium autotrophicum* HRM2	Marietou et al. 2018
Sulfate permease-like transporter, MFS superfamily	AET67377.1	*Desulfosporosinus orientis* DSM 765	Marietou et al. 2018
Sulphate transporter	ABO50513.1	*Desulfotomaculum reducens* MI-1	Marietou et al. 2018
**Group B**			
Putative sulfate transporter	CAE07354.1	*Synechococcus* sp. WH 8102	Price et al. 2004
Putative sulfate transporter	CAE21074.1	*Prochlorococcus marinus* str. MIT 9313	Price et al. 2004
Sulfate permease	NP_875196.1	*Prochlorococcus marinus* subsp. marinus str. CCMP1375	Price et al. 2004
Putative sulfate transporter	CAE19103.1	*Prochlorococcus marinus* subsp. pastoris str. CCMP1986	Price et al. 2004
SulP family inorganic anion transporter	WP_007304931.1	*Crocosphaera watsonii*	Price et al. 2004
Sulfate permease	BAB77999.1	*Nostoc* sp. PCC 7120	Price et al. 2004
slr1229	BAA17514.1	*Synechocystis* sp. PCC 6803	Price et al. 2004
SulP family inorganic anion transporter	WP_010637017.1	*Acidithiobacillus thiooxidans*	This study
SulP family inorganic anion transporter	WP_031570017.1	*Acidithiobacillus thiooxidans*	This study
Putative sulfate transporter ybaR	CDQ09458.1	*Acidithiobacillus ferrivorans* CF27	This study

Sulphate transporter	AEM47035.1	*Acidithiobacillus ferrivorans* SS3	This study
Sulfate permease SulP family	2507073582 (IMG/M)	*Hydrogenovibrio thermophilus* JR2	Scott et al. 2018
SulP family inorganic anion transporter	WP_028989891.1	*Thermithiobacillus tepidarius*	This study
**Group C**			
Sulfate permease SulP family	2507074344 (IMG/M)	*Hydrogenovibrio thermophilus* JR2	Scott et al. 2018
SulP family inorganic anion transporter	WP_013817236.1	*Methylomonas methanica*	Boden et al. 2011
SulP family inorganic anion transporter	WP_010960719.1	*Methylococcus capsulatus*	Ward et al. 2004
Carbonic anhydrase	WP_001024278.1	*Leptospira interrogans*	Felce and Saier, 2004
Sulfate transporter family protein	ACK80903.1	*Acidithiobacillus ferrooxidans* ATCC 23270	This study
Sulphate transporter	ACH82717.1	*Acidithiobacillus ferrooxidans* ATCC 53993	This study
Sulphate transporter	AEM48821.1	*Acidithiobacillus ferrivorans* SS3	This study
Sulphate transporter	CDQ10777.1	*Acidithiobacillus ferrivorans* CF27	This study
SulP family inorganic anion transporter	WP_006893903.1	*Methylobacter tundripaludum*	Svenning et al. 2011
Transmembrane carbonic anhydrase	NP_217790.1	*Mycobacterium tuberculosis* H37Rv	Felce and Saier, 2004
STAS domain-containing protein	WP_002615401.1	*Stigmatella aurantiaca*	Huntley et al. 2011
**Group D**			

Bicarbonate transporter, BicA	ACB00349.1	*Synechococcus* PCC 7002	Price et al. 2004
Low affinity sulfate transporter	BAL36634.1	*Synechocystis* sp. PCC 6803 substr. PCC-P	Price et al. 2004
Putative sulfate transporter	CAE08039.1	*Synechococcus* sp. WH 8102	Price et al. 2004
SulP family inorganic anion transporter	WP_011610814.1	*Trichodesmium erythraeum*	Price et al. 2004
SulP family inorganic anion transporter	WP_007306214.1	*Crocosphaera watsonii*	Price et al. 2004
Sulfate transporter/antisigma-factor antagonist STAS	ABA24816.1	*Trichormus variabilis* ATCC 29413	Price et al. 2004
Permease protein of sulfate transporter	BAC09698.1	*Thermosynechococcus elongatus* BP-1	Price et al. 2004
Low affinity sulfate transporter	BAL36690.1	*Synechocystis* sp. PCC 6803 substr. PCC-P	Price et al. 2004
Sulfate permease family protein	BAB73261.1	*Nostoc* sp. PCC 7120	Price et al. 2004
Sulfate permease	AAP99288.1	*Prochlorococcus marinus* subsp. marinus str. CCMP1375	Price et al. 2004
Putative sulfate transporter	CAE21389.1	*Prochlorococcus marinus* str. MIT 9313	Price et al. 2004
Putative sulfate transporter	CAE18673.1	*Prochlorococcus marinus* subsp. pastoris str. CCMP1986	Price et al. 2004
Putative sulfate transporter	CAE08451.1	*Synechococcus* sp. WH 8102	Price et al. 2004
**Group E**			

Sulphate transporter	ACC81271.1	*Nostoc punctiforme* PCC 73102	Felce and Saier, 2004
Sulfate transporter	ABM29712.1	*Desulfovibrio vulgaris* DP4	Marietou et al. 2018
Sulfate permease	WP_028989076.1	*Thermithiobacillus tepidarius*	This study
Putative sulfate transporter YchM	ABJ00623.1	*Escherichia coli* APEC O1	Moraes and Reithmeier 2012
C4-dicarboxylic acid transporter	BAB35134.1	*Escherichia coli* O157:H7 str. Sakai	Moraes and Reithmeier 2012
Sulphate transporter	ABO50171.1	*Desulfotomaculum reducens* MI-1	Marietou et al. 2018

References:

Boden R, Cunliffe M, Scanlan J, Moussard H, Kits KD, Klotz MG, Jetten MS, Vuilleumier S, Han J, Peters L, Mikhailova N, Teshima H, Tapia R, Kyrpides N, Ivanova N, Pagani I, Cheng JF, Goodwin L, Han C, Hauser L, Land ML, Lapidus A, Lucas S, Pitluck S, Woyke T, Stein L, Murrell JC. Complete genome sequence of the aerobic marine methanotroph Methylomonas methanica MC09. *J Bacteriol*. 2011;193(24):7001-2.

Felce J, Saier M. Carbonic anhydrases fused to anion transporters of the SulP family: evidence for a novel type of bicarbonate transporter. J Mol Microbiol Biotechnol. 2004;8(3):169-76.

Marietou A, Røy H, Jørgensen BB, Kjeldsen KU. Sulfate transporters in dissimilatory sulfate reducing microorganisms: A comparative genomics analysis. *Front Microbiol.* 2018; 9: 309.

Moraes TF, Reithmeier RA. Membrane transport metabolons. *Biochim Biophys Acta (BBA) – Biomembranes* 2012; 1818(11)**:** 2687-2706.

Price GD, Woodger FJ, Badger MR, Howitt SM, Tucker L. Identification of a SulP-type bicarbonate transporter in marine cyanobacteria. *Proc Natl Acad Sci U S A* 2004; 110 (52):18228-18233.

Scott KM, Leonard JM, Boden R, Chaput D, Dennison C, Haller E, Harmer TL, Anderson A, Arnold T, Budenstein S, Brown R, Brand J, Byers J, Calarco J, Campbell T, Carter E, Chase M, Cole M, Dwyer D, Grasham J, Hanni C, Hazle A, Johnson C, Johnson R, Kirby B, Lewis K, Neumann B, Nguyen T, Nino Charari J, Morakinyo O, Olsson B, Roundtree S, Skjerve E, Ubaldini A, Whittaker R. Diversity in CO_2-concentrating mechanisms among chemolithoautotrophs from the genera *Hydrogenovibrio*, *Thiomicrorhabdus*, and *Thiomicrospira*, ubiquitous in sulfidic habitats worldwide. *Appl and Environ Microbiol*. 2018; AEM.02096-18.

Huntley S, Hamann N, Wegener-Feldbrügge S, Treuner-Lange A, Kube M, Reinhardt R, Klages S, Müller R, Ronning CM, Nierman WC, Søgaard-Andersen L. Comparative genomic analysis of fruiting body formation in Myxococcales. *Mol Biol Evol* 2011; 28 (2):1083–1097.

Svenning MM, Hestnes AG, Wartiainen I, Stein LY, Klotz MG, Kalyuzhnaya MG, Spang A, Bringel F, Vuilleumier S, Lajus A, Médigue C, Bruce DC, Cheng JF, Goodwin L, Ivanova N, Han J, Han CS, Hauser L, Held B, Land ML, Lapidus A, Lucas S, Nolan M, Pitluck S, Woyke T. Genome sequence of the Arctic methanotroph *Methylobacter tundripaludum* SV96. *J Bacteriol*. 2011;193(22):6418-9.

Szklarczyk D, Morris JH, Cook H, Kuhn M, Wyder S, Simonovic M, Santos A, Doncheva NT, Roth A, Bork P, Jensen LJ, von Mering C. The STRING database in 2017: quality-controlled protein-protein association networks, made broadly accessible. *Nucleic Acids Res*. 2017;45(D1):D362-D368.

Ward N, Larsen Ø, Sakwa J, Bruseth L, Khouri H, Durkin AS, Dimitrov G, Jiang L, Scanlan D, Kang KH, Lewis M, Nelson KE, Methé B, Wu M, Heidelberg JF, Paulsen IT, Fouts D, Ravel J, Tettelin H, Ren Q, Read T, DeBoy RT, Seshadri R, Salzberg SL, Jensen HB, Birkeland NK, Nelson WC, Dodson RJ, Grindhaug SH, Holt I, Eidhammer I, Jonasen I, Vanaken S, Utterback T, Feldblyum TV, Fraser CM, Lillehaug JR, Eisen JA. Genomic insights into methanotrophy: the complete genome sequence of *Methylococcus capsulatus* (Bath). *PLoS Biol*. 2004; 2(10):e303.
