# Supplementary material for: Effect of CO2 Concentration on Uptake and Assimilation of Inorganic Carbon in the Extreme Acidophile Acidithiobacillus ferrooxidans
Source: Front Microbiol. 2019 Apr 4;10:603. doi: 10.3389/fmicb.2019.00603 (PMC6458275; doi:10.3389/fmicb.2019.00603)
Supplement: Supplementary file 4 [file Data_Sheet_4.PDF]

Effect of CO_2 Concentration on Uptake and Assimilation of Inorganic Carbon in the Extreme Acidophile *Acidithiobacillus ferrooxidans*

Mario Esparza, Eugenia Jedlicki, Carolina González, Mark Dopson, and David Holmes

SUPPLEMENTAL FIGURE S4

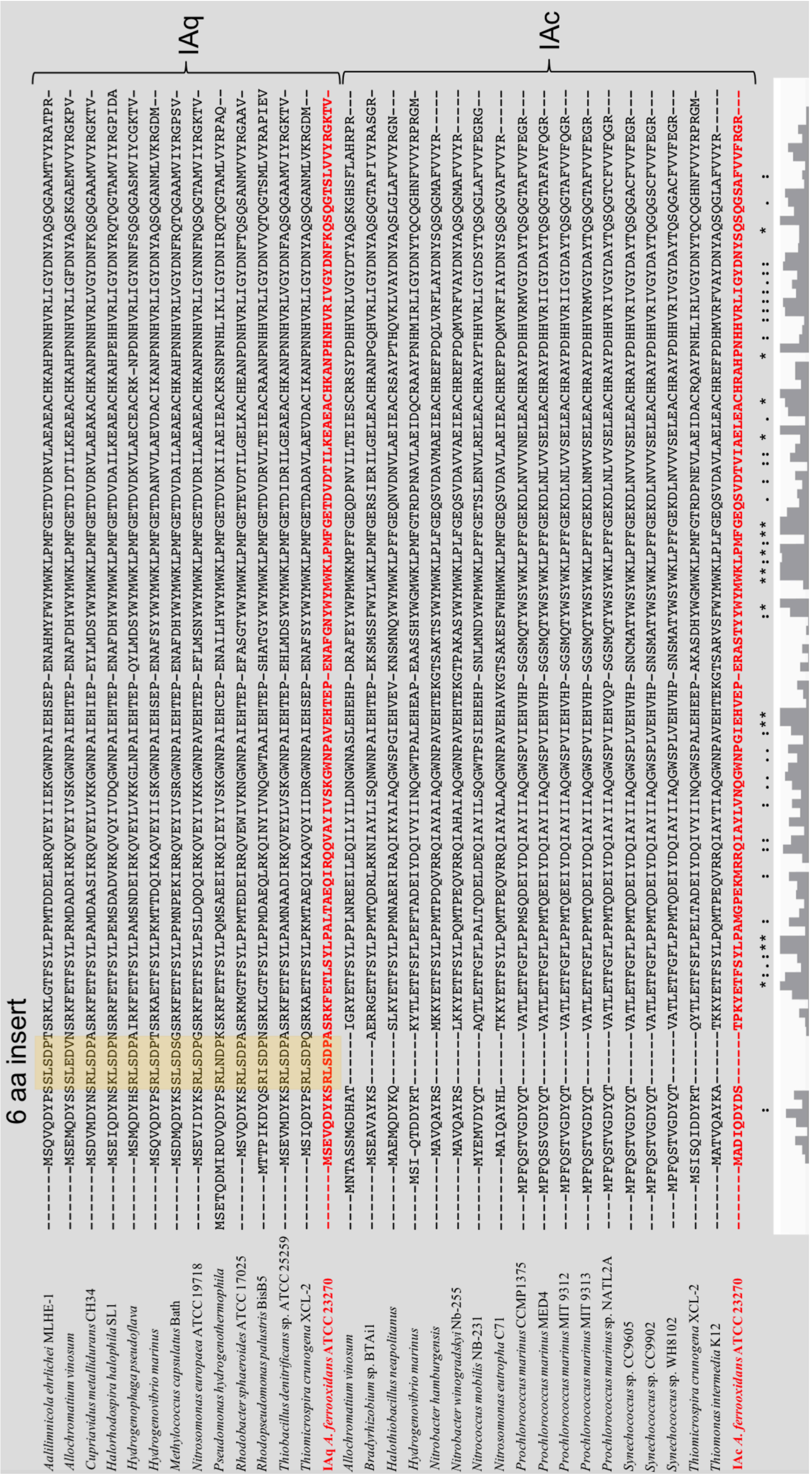

Six amino acid sequence insertion detected in Form IAq of the small subunit of RubisCo Form I of *Acidithiobacillus ferrooxidans* ATCC 23270 but not found in Form IAc of *A. ferrooxidans*

ATCC 23270. The two *A. ferrooxidans* sequences are highlighted in red and are compared to various cyanobacteria and proteobacteria Form IAq and IAc sequences obtained from Badger and Bek, 2008. Sequences were aligned with MAFFT with accurate option L-INS-i (Katoh and Standley, 2013; Nakamura et al., 2018). The overall degree of amino acid sequence conservation is shown at the bottom of the figure.

References:

Badger, M.R and Bek E.J., Multiple Rubisco forms in proteobacteria: their functional significance in relation to CO_2 acquisition by the CBB cycle, *Journal of Experimental Botany*, Volume 59, Issue 7, 1 May 2008, Pages 1525–1541.

Katoh, K., and Standley, D.M. (2013). MAFFT multiple sequence alignment software version 7: improvements in performance and usability. *Mol Biol Evol* 30**,** 772-780.

Nakamura, T., Yamada, K.D., Tomii, K., and Katoh, K. (2018). Parallelization of MAFFT for large-scale multiple sequence alignments. *Bioinformatics* 34**,** 2490-2492.
